# Mobile Attention Bias Modification Training Is a Digital Health Solution for Managing Distress in Multiple Sclerosis: A Pilot Study in Pediatric Onset

**DOI:** 10.3389/fneur.2021.719090

**Published:** 2021-07-28

**Authors:** Leigh Charvet, Allan George, Hyein Cho, Lauren B. Krupp, Tracy A. Dennis-Tiwary

**Affiliations:** ^1^Department of Neurology, NYU Grossman School of Medicine, New York, NY, United States; ^2^Department of Psychology, The Graduate Center, The City University of New York, New York, NY, United States; ^3^Department of Psychology, Hunter College, The City University of New York, New York, NY, United States

**Keywords:** multiple sclerosis, pediatric onset multiple sclerosis, pediatric neurology, attention bias modification training, anxiety, distress, telehealth, digital therapeutic

## Abstract

**Introduction:** Emotional health is important dimension of care for patients living with pediatric onset multiple sclerosis (POMS), but few options are available for stress and anxiety reduction. The high burden of interventions requiring regular in person and onsite visits for treatment are less feasible. Attention bias modification training (ABMT) is effective for anxiety reduction in adult and adolescent populations. We tested the feasibility and preliminary efficacy of ABMT delivered through a mobile gamified version as a digital emotional health tool for patients with POMS.

**Methods:** Participants with POMS were consecutively recruited from the NYU Langone Pediatric MS Care Center and enrolled to complete a 1-month intervention with use of the Personal Zen ABMT app on their mobile personal device. Feasibility was evaluated by use of the 1-month intervention and efficacy was measured by changes in depression, anxiety, and affect.

**Results:** A total *n* = 35 patients with POMS were enrolled in the study (*M*_*age*_ = 17.7, *SD* = 2.2 years, range 14–23). Feasibility criteria were met with 74% completing the full intervention time, and 100% of the sample completing at least 50% of targeted intervention use. Initial efficacy was found for a reduction in negative affect from baseline to intervention end [*M* = 22.88, *SD* = 9.95 vs. *M* = 19.56, *SD* = 7.37; *t*_(33)_ = 2.47, *p* = 0.019]. Anxiety also significantly decreased from pre to post-intervention in adults [*M* = 11.82, *SD* = 9.90 vs. *M* = 7.29, *SD* = 7.17; *t*_(16)_ = 3.88, *p* = 0.001] and youth [*M* = 51.14, *SD* = 19.66 vs. *M* = 40.86, *SD* = 27.48; *t*_(13)_ = 3.17, *p* = 0.007].

**Conclusion:** Mobile ABMT with the Personal Zen app is a feasible and accessible digital emotional health tool for patients with POMS and may have broader application for managing distress across chronic neurological conditions.

## Introduction

For those living with chronic neurological conditions such as multiple sclerosis (MS), psychological distress marked by anxiety and/or depression are frequent concerns across the lifespan (1, 2). Multiple sclerosis is a chronic and progressive demyelinating disorder of the central nervous system. Its earlier stages are typically defined by exacerbations of acute neuroinflammation and neurological deficit, followed by relative recovery (3). Multiple sclerosis is a lifelong disorder and without cure, but can be managed with disease modifying therapies (DMTs) and symptomatic treatments.

In MS, anxiety disorders are highly prevalent, affecting 29% of individuals during their lifetime and resulting in reduced occupational functioning and other societal costs (2, 4). While onset can occur throughout the lifespan, pediatric onset (<18 years of age) is considered a rare disorder of childhood, representing approximately 5% of all MS cases (5). Pediatric onset multiple sclerosis (POMS) shares many features with adult onset, but differs with increased frequency in exacerbations and also more robust recovery (6). The risk for psychological distress is especially pronounced for these younger MS patients diagnosed with the lifelong neurological condition, and in combination with the general higher rates of anxiety in the adolescents and young adult population in general (7).

Anxiety and associated stress-related problems are a particularly important target of intervention for patients with POMS (8, 9), with more than one-third reporting significant psychological concerns (8, 9). The adjustment to diagnosis and the experience of illness is particularly significant with childhood onset (7). Psychological distress can be more pronounced when MS results in functional declines, and particularly with cognitive involvement (9–11). However, even when neurologically intact, patients with POMS can be associated with increased problems with the psychological aspects of the disease and its resulting impact on academic and social functioning (8, 9). In addition, these younger patients share the broader psychological vulnerability associated with the current focus on social media and the often inflammatory web-based content, often inaccurately reflecting life with MS (12, 13).

Unfortunately, there is a major unmet need for access to mental health services in general (14, 15), including for the many patients living with neurological or medical conditions, Specifically for younger patients with neurological conditions who have ongoing, yet subclinical threshold symptoms do not have many options for management of emotional health (16). Due to the low prevalence of disease, many patients with POMS receive care at tertiary care centers geographically distant from their home. This distance restricts access to onsite therapies due to logistical barriers with transportation are further complicated by the necessity of parents' presence for onsite interventions. Both the need for management options and the barriers to onsite treatment access were further highlighted for patients with POMS in the context of the COVID-19 pandemic (17). A digital health-based solution for emotional health can overcome these challenges.

Digital therapeutics represent a rapidly emerging option for intervention, offering mobile access to treatment. Digital health interventions can include many advantages for reaching many more patients in need, providing more timely intervention, and also to deliver intervention in the amount and frequency often needed for optimal benefit (18). A particularly promising, focused intervention strategy targets anxiety-related attention bias (AB), a cognitive mechanism in anxiety that AB refers to selective and exaggerated attention toward threatening information and stimuli, and has been shown to play a significant role in the etiology and maintenance of anxious pathology in children, adolescents, and adults (19–25).

Strong evidence base has driven significant enthusiasm for the development of interventions that directly target the reduction of AB (26–29). One established intervention is attention bias modification training (ABMT). ABMT is brief, cost-effective, safe, and well-tolerated. Therefore, it may represent an optimal anxiety- and stress-reduction intervention for youth experiencing chronic illness, for whom intensive treatments may be too time-consuming and for whom medication-based approaches carry significant risk. ABMT is a computerized intervention designed to reduce AB among participants evidencing symptoms across the broad spectrum of anxiety and stress-related disorders by repeatedly directing attention away from threat-relevant cues using modified dot probe and visual search paradigms (20, 26, 30–33).

For this study, we selected the first mobile version of ABMT to be developed as a digital health intervention. This mobile version is a downloadable iOS Application called “Personal Zen” and has been modified with the aim of increasing accessibility of the program and in an effort to make the task more enjoyable and engaging than the traditional lab-based protocols. This gamified version offers a more accessible and engaging experience than the traditional protocols. Provided as a mobile application or “app,” it takes the core components of the gold-standard ABMT protocol (the dot probe task) and puts them in the context of an appealing exercise, incorporating video game-like features such as animated characters and sound effects (34). Like traditional ABMT, attention is still systematically redirected away from threat-relevant stimuli (angry faces). Four randomized clinical trials of the app document that between a single session and 4 weeks of use effectively reduces biobehavioral and cognitive indices of anxiety and stress, as well as AB measured via the dot probe (34–36). These data demonstrate that the app is an effective delivery system for ABMT.

The emergent need for digital emotional health tools for patients with POMS was heightened in the context of the COVID-19 pandemic, where both overall emotional distresses significantly increased while access to onsite care was restricted. Here, during the course of the pandemic, we evaluated the feasibility and preliminary efficacy of ABMT using Personal Zen in a sample of patients with POMS.

## Materials and Methods

### Participants

Participants ages 12–24 years with a confirmed diagnosis of POMS, as defined by the 2013 International Pediatric Multiple Sclerosis Study Group (IPMSSG) criteria (37) and the 2010 McDonald criteria (3), were recruited for this study to test the feasibility of use of the Personal Zen app. Participants were not specifically recruited on the basis of emotional health status, and told that the purpose of the study was to evaluate a mobile (phone) application aimed at reducing anxiety and stress among younger patients with MS. Participants were consecutively recruited during routine outpatient or telemedicine visits at the pediatric MS center through the NYU Langone MS Comprehensive Care Center in New York, NY between June 2020 and February 2021. Potential participants were identified by the treating neurologist (Dr. Krupp) and screened for eligibility (Table 1) onsite, or via a telemedicine visit by a trained member of the study team at the conclusion of their clinic visit. This study was approved by the institutional review board of NYU Langone Health.

**Table 1:** Inclusion and exclusion criteria.

**Inclusion criteria**	**Exclusion criteria**
Ages 12–24	Previous report of an IQ <70
Confirmed diagnosis of multiple sclerosis with onset <17 years and 11 months	Wide-range achievement test-fourth edition (38) Reading Subtest standard score <85
Followed at NYU Multiple Sclerosis Comprehensive Care Center	Non-English speaking, learned English in the past 3 years, or learned English after the age of 12 years
Access to a mobile device with iOS devices	Not willing to comply with all study procedures
	Insufficient visual and motor ability to operate the intervention and assessments

Participants received payment for each study milestone completed (baseline/weekly evaluations/end of study) for up to $72.00 total for participation across the study.

### Study Overview

Once enrolled, participants completed baseline measures and were instructed on access of the Personal Zen application on their personal device. They then completed a 1-month period of directed daily use, with weekly remote assessments. At intervention end, baseline outcome measures were repeated.

### Measures

#### Baseline Demographic and Clinical Features

Baseline demographic and MS disease measures were recorded (Table 2) and the clinical rating of neurologic disability, the Expanded Disability Status Scale or EDSS (39), was administered by the treating neurologist. We also included a screening measure of MS-related cognitive functioning administered clinically, the Symbol Digit Modalities Test or SDMT (40).

**Table 2:** Demographic and clinical features (*N* = 35).

**DEMOGRAPHIC FEATURES**	
Age (*M, SD*, range)	17.69, 2.21, 14–23 years
Gender (%)	80% Female 20% Male
Hispanic/Latino (%)	34%
Race (%)	40% Black/African American 6% Asian 34% White 20% Other
**CLINICAL FEATURES**	
WRAT-4 reading (SS-*M, SD*)	105.29 (12.81)
SDMT (raw score-*M, SD*)	59.31 (13.26)
EDSS (median, range)	0.0 (0.0–3.5)
MS disease duration (*M, SD* years)	2.86 (1.91)

#### Feasibility and Evaluation of Intervention

Feasibility was defined as at least 50% of sample completing at least 50% of targeted use of the intervention across the 1-month study period.

At the end of the study, participants also completed a rating of their experience with the intervention. The Debriefing Questionnaire was created for this study and included five open-ended questions addressing the user's experience with the Personal Zen application.

#### Baseline Attention Bias

##### The Dot Probe

The dot probe task (19, 24) was administered at baseline of the study to measure AB. Due to a change in the method of study administration from in-person to remote due to COVID-19-related restrictions in the recruitment year 2020, out of 35 participants, 15 completed in-person dot probe task (42.9%), and 20 completed online dot probe task (57.1%).

Both versions of the dot probe task followed parameters of the Tel-Aviv University/National Institute of Mental Health protocol. Stimuli for the dot probe task are pictures of 20 different individuals (10 males, 10 females) from the NimStim stimulus set (41) with one female taken from the Matsumoto and Ekman (42) set. For in-person participants, stimuli were programmed and presented using E-Prime version 2.0 (43). For remotely enrolled participants, the same task was presented via an online platform.

During each trial, two facial stimuli were presented, either angry-neutral face pairs or neutral–neutral face pairs of the same actor. The facial stimuli were presented above and below a fixation cross, with 14 mm between them. The task consisted of 120 trials [80 threat (angry faces) and neutral facial stimuli (TN) and 40 non-threat both neutral facial stimuli (NN)]. Each trial comprised of; (a) 500 ms fixation, (b) 500 ms face-pair cue, which then disappears, (c) probe in the former location of one of the facial stimuli until a response is made via the left or right arrow key to indicate the direction in which the arrow is pointing, and (d) 500 ms inter-trial interval. Participants were asked to respond as quickly and as accurately as possible whether the arrow was pointing to the left or the right. For the task, probes were equally likely to appear on the top or bottom, in the location of the angry or neutral face cues and pointing to the left or the right.

##### Quantifying Attention Bias

Attention bias was measured via the dot probe task. Dot probe trials with incorrect responses were excluded from further processing and analyses. Responses for each individual were removed if they were faster than −3 *SD* and slower than +3 *SD* from the individual's mean to normalize the distribution, and is a standard practice in reaction time based data. In addition, all participants had an accuracy rate of 85% or above. First, a threat bias score was computed to quantify overall attention capture by threat, as the average RTs for neutral probes in TN trials minus RTs for angry probes in TN trials. In addition, a vigilance score (automatic, bottom-up attention) was calculated, as the average RTs for neutral probes in the NN trials minus the average RTs for threat probes in TN trials. Lastly, to quantify the more effortful top-down inhibition of attention, a difficulty disengaging score was computed as the average RTs for neutral probes in TN trial minus RTs for neutral probes in the NN trials.

#### Self-Reported Affect, Anxiety, and Depression Symptoms

Participants completed questionnaires at pre-intervention baseline, weekly during the 1-month intervention (week 2), and again at the end of study (post-intervention). Only pre- and post-intervention data are reported below. Questionnaires were completed with REDCap or MyCap.

##### Positive and Negative Affect

All participants completed the Positive and Negative Affect Schedule [PANAS; (44)], a self-report questionnaire consisting of two 10-item scales, one assessing positive affect (e.g., Interested, Excited, Strong) and one assessing negative affect (e.g., Distressed, Upset, Guilty). Each item is rated on a 5-point scale with 1 indicating “not at all” to 5 indicating “very much.” The PANAS shows high internal consistency and reliability.

##### Anxiety and Depressive Symptoms

The following questionnaires were administered to participants younger than 18 years of age:

*Child Depression Inventory 2* (CDI 2) (45) is a self-report 28-item measure designed to assess the severity of depressive symptoms in children and adolescents over the previous 2-week period. Questions are rated on a 0–2 scale, and the total scores of 0–14 is considered minimal symptoms, 15–19 is mild, 20–24 is moderate, and 25–56 is severe depressive symptoms. The CDI 2 has high internal consistency and reliability.
*Multidimensional Anxiety Scale for Children Second Edition Parent Version* (MASC 2-P) (46) is a 50-item measure of anxiety symptoms in children and adolescents rated on a scale of 0–3, completed by parents. In addition to a total score, subscales include separation anxiety, generalized anxiety, social anxiety, and physical arousal. The MASC 2-P has high internal consistency and reliability. Higher scores indicate more severe and/or greater number of symptoms, with total scores higher than 65 classified as clinically significant impairment.

Participants older than 18 years of age completed the following questionnaires:

*Beck Depression Inventory* (BDI-II) (47) is a self-report 21-item questionnaire designed to assess depressive symptoms over the previous 2-week period. Questions are rated on a 0–3 scale, with scores ranging from 0 to 63. The total scores of 0–13 is considered minimal symptoms, 14–19 is mild, 20–28 is moderate, and 29–63 is severe depressive symptoms. The BDI has high internal consistency and reliability.
Beck Anxiety Inventory (BAI) (48) is a self-report 21-item questionnaire designed to assess symptoms of anxiety over the past month including cognitive, behavioral, and physiological arousal. Questions are rated on a 4-point scale from 0 indicating “not at all” to 3 indicating “severely” (e.g., “unsteady,” “nervous”). The total scores of 0–7 is considered minimal symptoms, 8–15 is mild, 16–25 is moderate, and 26–63 is severe anxiety symptoms. The questionnaire has a clinical score cut-off of 16 and above. The BAI has high internal consistency and reliability.

#### Intervention-Mobile, Gamified Attention Bias Modification Training

All participants received the active version of the mobile app training based on a modified the dot probe task (i.e., ABMT), commercially available under the name Personal Zen. At the baseline visit, participants were instructed to download the iOS Personal Zen application on their personal device. Participants used their mobile device (e.g., iPhone) to practice the app to ensure understanding under the guidance of the experimenter [see (36)].

The following instructions were provided to each participant: “In this attention training app, two animated faces will appear on the screen. Shortly after, they will burrow into a hole. One of them will cause a path to rustle behind it. With your finger, trace the path of the rustling grass, beginning from the burrow. Trace the grass as smoothly, quickly, and accurately as possible. At no point should you feel rushed, you should be comfortable.”

Participants completed one practice round, and the experimenter stayed in the room during the practice round to answer any questions about the app. Two animated faces (sprites), one showing an angry expression and one showing a neutral/mildly pleasant expression, appeared simultaneously on the screen for 500 ms on every trial. Following the presentation, both sprites simultaneously “burrowed” into the field [see (36) for images of the app, (35)]. Next, a path of flowers and leaves appeared in the location of the neutral face for every trial. The path remained until participants responded by tracing it starting from the point at which the sprite burrowed into the field.

Participants were instructed to play the mobile app for a minimum of four times per week, 10–15 min a session, for 4 weeks (minimum of 16 sessions). Each session consisted of approximately 40–45 app rounds (varied based on user speed) with 12 trials per round. Number of training trials were consistent with previously documented effective “dosages” of the app (34–36).

Participants were asked to keep track of which days they used the App on the Personal Zen Log via REDCap or MyCap (Mobile REDCap Application). This application did not collect PHI and was free to use for participants.

## Results

A total of *n* = 35 participants were enrolled, with demographic and clinical features show in Table 2.

### Feasibility and Acceptability of Intervention

Of the *n* = 35 participants, all (100%) met feasibility criteria of intervention use of 50% of targeted amount (at least eight times over study). Further, *n* = 26/35 (74%) completed the fully targeted use (at least four times per week over 4 weeks).

During the debriefing interview at the end of the 4-week intervention period, 67% of participants reported gaining benefit from using Personal Zen. Many users provided positive comments, including:

“*…using the app did calm me down while I was stressed or overwhelmed. It gave me an opportunity to wind down”*

“*I thought the app provided a peaceful distraction, at least temporarily, from what I was dealing with at any particular time.”*

“*I looked forward to using it whenever my day was particularly stressful.”*

“*Overall, I thought it was useful in moderately reducing my stress and I will continue using it after the study ends.”*

### Preliminary Efficacy for Distress Reduction

Descriptive statistics for self-report of NA and PA, anxiety symptoms, and depressive symptoms are presented in Table 3. Attention bias measured at baseline yielded three metrics: threat bias (*M* = −7.70, *SD* = 28.04), vigilance (*M* = −7.26, *SD* = 41.74), and difficulty disengaging (*M* = −0.44, *SD* = 31.99).

**Table 3:** Descriptive statistics for self-report of affect, anxiety, and depression.

**Questionnaire**	**Baseline**	**End of Treatment**
	***M (SD) Min–Max***	***M (SD) Min–Max***
**AFFECT**		
*PANAS—positive affect*	29.09 (7.55) 13–44	28.59 (8.84) 10–45
*PANAS—negative affect*	22.88 (9.95) 11–48	19.56 (7.37) 10–37
**ANXIETY SYMPTOMS**		
*BAI*	11.82 (9.90) 1–32	7.29 (7.17) 0–25
*MASC 2-P*	51.14 (19.66) 14–103	40.86 (27.48) 4–102
**DEPRESSION SYMPTOMS**		
*BDI*	15.59 (9.55) 2–33	12.47 (13.02) 0–39
*CDI 2*	26.06 (2.72) 22–30	26.25 (3.73) 17–32

*PANAS, Positive and Negative Affect Schedule; BDI, Beck's Depression Inventory; CDI 2, Children's Depression Inventory; BAI, Beck's Anxiety Inventory; MASC-2 P, Multidimensional Scale for Children 2^nd^ Edition-Parent*.

For the measures of anxiety and depressive symptoms, the following percentage of participants fell in a clinical range at baseline: CDI 2: 18/18 participants at or exceeding the mild level of depression scoring above 15 (100%); MASC 2-P: 5/18 participants at or exceeding the score of 65, which is considered to be clinically significant impairment (28%); BDI: 9/17 participants at or exceeding the mild level of depression scoring above 14 (53%); and BAI: 12/17 participants at or exceeding mild level of depression scoring above 8 (71%).

Table 4 presents correlations among all study variables at pre-intervention baseline (NA, anxiety, depression, and AB scores). Overall, NA, anxiety (BAI), and depression (BDI) were significantly positively correlated (*r*s < 0.58, *p*s < 0.015).

**Table 4:** Correlations between the AB scores and symptoms at baseline.

	**1**	**2**	**3**	**4**	**5**	**6**	**7**	**8**
1.NA	–	0.64^**^	−0.081	0.58^*^	−0.40	0.30	0.39^*^	−0.25
2.BAI		–	.	0.74^**^	.	−0.057	−0.17	0.12
3.MASC 2-P			–	.	0.40	−0.42	−0.59^**^	0.51^*^
4.BDI				–	.	0.081	−0.24	0.34
5.CDI 2					–	−0.020	−0.16	0.20
6.TB						–	0.64^**^	0.038
7.VIG							–	−0.74^**^
8.DIS								–

*Each “.” denotes no correlation due to a lack of overlap in variables. NA, Negative Affect; BAI, Beck's Anxiety Inventory; MASC 2-P, Multidimensional Scale for Children II, Parent Report; BDI, Beck's Depression Inventory; CDI 2, Children's Depression Inventory; TB, threat bias; VIG, vigilance; DIS, difficulty disengaging.*

* *p < 0.05*

** *p < 0.01*.

Participants were instructed to use the mobile app for a minimum of 16 sessions over the course of their study participation. Self-reported use exceeded, on average, the minimum number of requested sessions (*M* = 17.37, *SD* = 5.30; *Min–Max*: 9–33; *Mode*: 16).

### Split-Half Reliability of Attention Bias Scores

Split-half reliability was examined for the dot probe task by creating mean RTs by experimental condition (neutral probes in TN trials, angry probes in TN trials, neutral probes in NN trials) and traditional AB scores (threat bias, vigilance, and disengaging), separately for even and odd trial for the dot probe task at baseline.

Even and odd mean RTs were all significantly positively correlated (*r*s > 0.92, all *p*s < 0.0001). In contrast, mean AB scores for even and odd trials did not significantly correlate (all *p*s > 0.05), consistent with prior literature (49–51).

### Main Analyses

To test the hypothesis that NA and symptoms of anxiety and depression will decrease from the pre- to post-intervention, and that PA will increase, we compared pre- and post-intervention assessments of affect (PANAS NA and PA subscales), anxiety (BAI and MASC 2-P), and depression (BDI and CDI 2) using paired samples *t*-tests.

**Figure 1:**
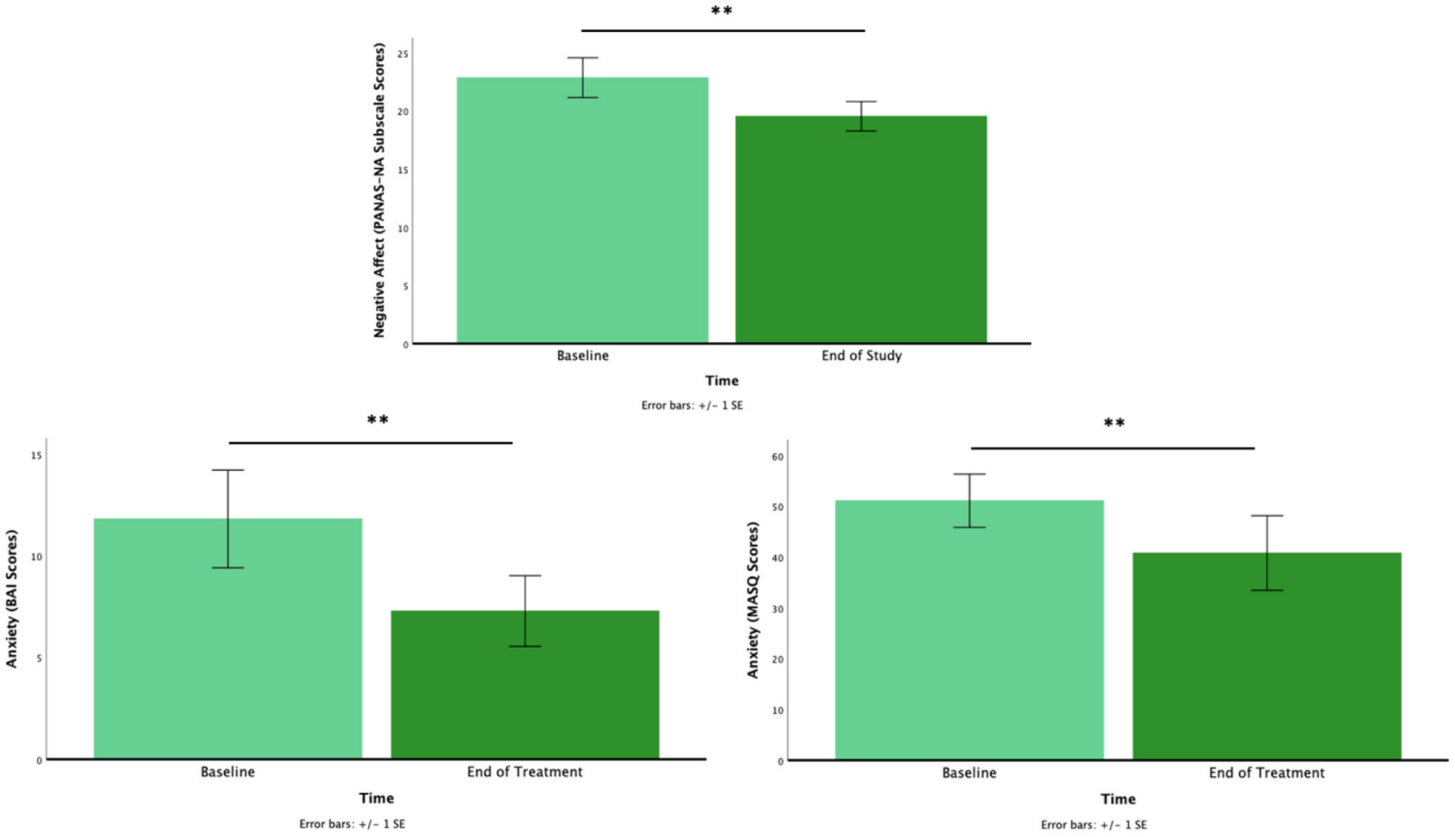
The pre- to post-intervention for negative affect (top), anxiety for adults (bottom left), and anxiety for youth (bottom right). *p < 0.05, **p < 0.01.

As predicted, NA decreased from pre-to post-intervention [*M* = 22.88, *SD* = 9.95 vs. *M* = 19.56, *SD* = 7.37; *t*_(33)_ = 2.47, *p* = 0.019]. Also, as predicted, anxiety significantly decreased from pre to post-intervention in adults [*M* = 11.82, *SD* = 9.90 vs. *M* = 7.29, *SD* = 7.17; *t*_(16)_ = 3.88, *p* = 0.001] and youth [*M* = 51.14, *SD* = 19.66 vs. *M* = 40.86, *SD* = 27.48; *t*_(13)_ = 3.17, *p* = 0.007; Figure 1]. No other comparisons reached significance (*p*s > 0.05).

Based on our main findings with NA, we conducted an exploratory analysis to examine if there were any differences between high anxiety (HA) vs. low anxiety (LA) individuals. We used baseline anxiety (i.e., pre-intervention BAI and MASC 2-P) for a median split to identify HA (*n* = 17) and LA (*n* = 13) to compare their treatment outcomes for NA. The results showed that the reduction of NA from pre- and post-intervention assessment was evident in the HA group only, [*M* = 24.65, *SD* = 7.89 vs. *M* = 20.88, *SD* = 6.56; *t*_(16)_ = 2.33, *p* = 0.033], but not in the LA group [*M* = 17.33, *SD* = 10.71 vs. *M* = 15.25, *SD* = 5.55; *t*_(11)_ = 0.90, *p* > 0.05].

### Exploratory Analyses

We tested the exploratory hypothesis that baseline AB scores (threat bias, vigilance, and difficulty disengaging, and RTs) would predict changes in NA and anxiety pre- to post-intervention. To do so, we conducted correlations between AB scores and difference scores (baseline minus at the end of treatment) for NA (sample as a whole) and anxiety (separately for teens and adults). Results indicated that vigilance was significantly intercorrelated with threat bias (*r* = 0.64, *p* < 0.001) and difficulty disengaging (*r* = −0.74, *p* < 0.001). However, AB scores were not significantly correlated with change in NA or anxiety measures (*r*s ranging from −0.034 to −0.33, all *p*s > 0.05). Correlations between RTs and NA and anxiety scores also did not reach significance (*r*s ranging from 0.07 to 0.12, all *p*s > 0.05).

## Discussion

Patients with POMS face not only distress related to ongoing disease activity and treatment, but the emotional adjustment to a diagnosis of a chronic and progressive disorder without cure. In addition to the risk for anxiety associated with adolescence and young adulthood in general, these patients are particularly vulnerable to increased emotional distress and resulting impact on their functioning and quality of life.

There remains a major unmet need for accessible and effective interventions for patients living with neurological conditions and including MS. Reflecting the focus and development of digital technologies to reach those living with MS (52, 53), digital emotional health tools can provide a solution. Here, we found the ABMT app, Personal Zen, to be feasible to reach a sample of patients with POMS with high fidelity to the intervention due to its convenience. In addition, we found a significant benefit of the intervention for reducing negative affect and anxiety symptoms among a sample not specifically recruited due to baseline emotional health status. In addition, the exploratory analysis with high and low baseline anxiety showed that those who exhibited high baseline anxiety gained more benefit with ABMT in reducing negative affect. While this finding is limited due to our small sample size, this could be informative for future studies. Further, while not recruited for this study based on emotional health status, we noted a high rate of depression in our sample and a resulting decrease in depressive symptoms with the intervention. This is consistent with prior studies on ABMT broadly (20, 54), and Personal Zen in particular (34, 36), highlighting the potential cross-diagnostic benefit of ABMT for the use in distress, which can present with increased anxiety or depression, or both (55, 56).

The next step for continued investigation is a controlled trial to study ABMT in this younger population of patients living with MS. It will be important to determine its specific therapeutic role, including whether those with clinical elevations of distress and anxiety can receive particular benefit. Further, it will be important to measure the corresponding changes in disease burden (e.g., symptom experience) and academic and social functioning. Finally, the impact of individual differences in AB prior to training remain unclear (57). Indeed, exploratory analyses from the current study failed to document significant correlations between baseline AB and ABMT effects on mood, although the unreliability of AB metrics and small sample size were limitations to adequately testing this exploratory hypothesis. Future research should examine additional and more reliable measures of AB including eye-tracking metrics, and neural indices of threat processing (35), as well as examine how changes in AB due to intervention lead to reduction on clinical measures such as anxiety and depression. In addition, studies directly comparing Personal Zen to other applications can inform as to whether the benefit is unique to its specific features or more general to ABMT across delivery methods.

Limitations to the study include the open-label design. While participants were recruited to evaluate the intervention for feasibility of use, the clinical benefit may have included placebo response. The small sample size prevented our ability to conduct multivariate analyses examining the role of symptom severity or other individual differences. Further research is needed to test whether the intervention is feasible for older individuals living with MS and generalization to those living with other chronic neurological conditions. However, given the overall rare subpopulation represented in this current study, as well as the overall acceptability and accessibility of the Personal Zen intervention, findings may be directly relevant to current clinical practice.

Psychological distress can adversely affect social-emotional well-being and other key metrics of functioning and perceived health in the context of MS and other chronic neurological conditions. Younger patients with MS may be at both increased risk for distress and face even greater access to interventions. We found that the digital emotional health intervention of mobile and gamified ABMT, delivered by the Personal Zen app, was both feasible for use and resulted in decreased negative affect and depressed mood. These results inform and support going forward with future investigations including randomized and controlled trial designs to of ABMT as digital emotional health intervention in both younger and older patients living with MS and other chronic neurological conditions.

## Product Information

Personal Zen is a gamified mobile app designed to deliver attention bias modification training (ABMT). Empirical evidence has shown that ABMT, specifically Personal Zen, can reduce anxiety-related attention bias, anxiety, and stress. Personal Zen is a product of Wise Therapeutics, Inc., and is commercially available.

## Data Availability Statement

The raw data supporting the conclusions of this article will be made available by the authors, without undue reservation.

## Ethics Statement

The studies involving human participants were reviewed and approved by NYU Langone IRB. Written informed consent to participate in this study was provided by the participants' legal guardian/next of kin.

## Author Contributions

LC and TD-T conceived of and designed the study. AG recruited and enrolled participants and collected data for analyses. LK provided neurological evaluation and recruited participants. AG, HC, TD-T, and LC contributed to the analyses and prepared the manuscript. All authors edited, reviewed, and approved of the manuscript for submission.

## Conflict of Interest

TD-T has equity in Wise Therapeutics, Inc., which owns Personal Zen, and is on the advisory board of Lil Space Inc. TD-T is an inventor, with IP under patent review, on a digital therapeutics system and cognitive training method related to Personal Zen. The remaining authors declare that the research was conducted in the absence of any commercial or financial relationships that could be construed as a potential conflict of interest.

## Publisher's Note

All claims expressed in this article are solely those of the authors and do not necessarily represent those of their affiliated organizations, or those of the publisher, the editors and the reviewers. Any product that may be evaluated in this article, or claim that may be made by its manufacturer, is not guaranteed or endorsed by the publisher.
